# Supplementary material for: Anemoside B4 Rectal Thermosensitive In Situ Gel to Treat Ulcerative Colitis by Overcoming Oral Bioavailability Barriers with Absorption Enhancer-Assisted Delivery
Source: Pharmaceutics. 2025 Oct 29;17(11):1400. doi: 10.3390/pharmaceutics17111400 (PMC12655041; doi:10.3390/pharmaceutics17111400)
Supplement: Supplementary file 1 [file pharmaceutics-17-01400-s001.zip › pharmaceutics-3877623-supplementary.pdf]

Supplementary materials

Figures

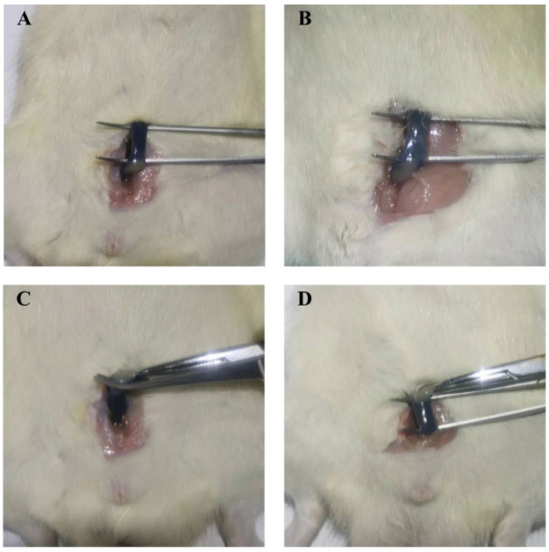

Figure S1. In vivo localization of HP-β-CD-AB4-ISG and SC-AB4-ISG in the rectum at (A, C) 0.5h and (B, D) 6h after rectal administration.

Tables

Table S1. Independent variables and their values in BBD-RSM.

Level	Independent variables and their values		
	A, P407 (%,w/w)	B, P188 (%,w/w)	C, HPMC (%,w/w)
-1	17	4	0.3
0	18.5	6	0.45
1	20	8	0.6

Table S2. BBD-RSM design and results ($\overline{x}$±*s*, *n*=6).

Number	Levels of variables			Actual values of variables			Observed
	A	B	C	P407/%	P188/%	HPMC/%	T/°C
1	0	0	0	18.50	6.00	0.45	30.60±0.36
2	0	0	0	18.50	6.00	0.45	32.00±0.44
3	0	-1	1	18.50	4.00	0.60	27.43±0.78
4	0	0	0	18.50	6.00	0.45	29.50±1.13

5	1	1	0	20.00	8.00	0.45	27.87±0.93
6	-1	1	0	17.00	8.00	0.45	38.00±0.78
7	-1	0	-1	17.00	6.00	0.30	35.93±0.87
8	0	0	0	18.50	6.00	0.45	31.37±0.85
9	0	1	1	18.50	8.00	0.60	32.67±0.81
10	-1	0	1	17.00	6.00	0.60	36.63±1.12
11	0	-1	-1	18.50	4.00	0.30	28.40±0.70
12	0	1	-1	18.50	8.00	0.30	31.63±0.15
13	1	0	-1	20.00	6.00	0.30	25.70±0.87
14	1	0	1	20.00	6.00	0.60	25.00±1.08
15	1	-1	0	20.00	4.00	0.45	22.93±0.81
16	0	0	0	18.50	6.00	0.45	30.27±0.21
17	-1	-1	0	17.00	4.00	0.45	32.50±0.85

Table S3. Regression coefficient and variance analysis of each factor

Error source	SS	f	S	F	p	Significance
Model	262.18	6	43.70	66.90	＜0.0001	**
A-P407	215.90	1	215.90	330.53	＜0.0001	**
B-P188	44.70	1	44.70	68.43	＜0.0001	**
C-HPMC	6.125×10^{-4}	1	6.125×10^{-4}	9.377×10^{-4}	0.9762	
AB	0.078	1	0.078	0.12	0.7362	
AC	0.49	1	0.49	0.75	0.4067	
BC	1.01	1	1.01	1.55	0.2421	
ABC	0.000	0				
Residual	6.53	10	0.65			
Lack of Fit	2.77	6	0.46	0.49	0.7920	
Pure Error	3.76	4	0.94			
Cor Total	268.71	16				

Note：$^{**}p$＜0.01，$^{*}p$＜0.05

Table S4. In vitro release curve fitting of in situ gels using porcine rectal mucosa.

Model	AB4-ISG	HP-β-CD-AB4-ISG	SC-AB4-ISG
Zero-order kinetic	Mt=14.27X+9.55<br>R^2=0.8520	Mt=13.88X+18.02<br>R^2=0.7227	Mt=14.87X+11.62<br>R^2=0.8065
First-order kinetic	Mt=90.66(1-$e^{0.40t}$)<br>R^2=0.9849	Mt=85.80(1-$e^{0.67t}$)<br>R^2=0.9836	Mt=91.63(1-$e^{0.46t}$)<br>R^2=0.9784
Higuchi kinetic	Mt=29.40$t^{1/2}$+19.70<br>R^2=0.8283	Mt=19.38$t^{1/2}$+8.637<br>R^2=0.8097	Mt=18.08$t^{1/2}$+5.581<br>R^2=0.8169

Table S5. Pharmacokinetic parameters of AB4 after a single administration ($\bar{x}$±*s*, *n*=6).

group	C_{max}	T_{max}	$t_{1/2}$	$MRT_{(0-t)}$	$AUC_{0-\infty}$	F/%
	ug/L	h	h	h	ug/L*h	
ig	1338.5±774.917	0.542±0.246	2.121±1.065	3.754±3.369*	3261.733±1500.954*	0.15
iv	23216.667±10400.657#	0.611±0.453	0.831±0.176	1.912±0.344	49591.614±14083.409	-
AB4-ISG	13526.667±13563.191	3±1.265*#	4.455±6.9*#	4.963±2.921*	40037.623±26461.423*	38.45
HP-β-CD-AB4-ISG	10983.333±2263.11a	2.125±2.328*#	1.591±0.907	3.821±1.742*	75029.45±87234.475*	72.05
SC-AB4-ISG	6870±5751.438#a	3±0.632*#	2.406±1.355*#	3.917±0.432*	62841.854±24092.819*	60.34

Note: *P<0.05 vs the IV group，#P<0.05 vs the IG group，aP<0.05 VS the AB4-ISG group.

$AUC_{(0\rightarrow\infty)}$, area under the plasma concentration time curve calculated using the trapezoidal rule from time 0 to infinity; SD, standard deviation; $t_{1/2}$, elimination half-life; C_{max}, peak plasma concentration; T_{max}, time to reach the peak plasma concentration; F, represent absolute bioavailability.
